# Addressing racism in the workplace through simulation: So much to unlearn

**DOI:** 10.3389/fresc.2023.1126085

**Published:** 2023-03-30

**Authors:** Moni Fricke, Debra Beach Ducharme, Allana Beavis, Priscilla Flett, Sarah Oosman

**Affiliations:** ^1^ College of Rehabilitation Sciences, University of Manitoba, Winnipeg, MB, Canada; ^2^ Global Health Division, Canadian Physiotherapy Association, Ottawa, ON, Canada; ^3^ Ongomiizwin Indigenous Institute of Health and Healing, University of Manitoba, Winnipeg, MB, Canada; ^4^ Community Therapy Services Inc., Winnipeg, MB, Canada; ^5^ School of Rehabilitation Science, University of Saskatchewan, Saskatoon, SK, Canada

**Keywords:** racism, anti-racism, reconciliation, virtual, simulation, evaluation, rehabilitation

## Abstract

**Introduction:**

Racism exists in the healthcare system and is a root cause of health inequities among Indigenous Peoples. When microaggressions of racism are carried out by healthcare providers, therapeutic trust may be broken and quality of care may be impacted. Anti-racism response training is considered best practice in recognizing and addressing racism. The objective of this study was to evaluate the impact of a virtual (synchronous) anti-racism response training workshop among a group of rehabilitation therapists from across Canada.

**Methods:**

A 90-minute virtual anti-racism simulation workshop for rehabilitation therapists was developed and delivered virtually four times across Canada between 2020 and 2021. Following an introduction and pre-briefing, role-playing among participants was used to address microaggressive Indigenous-specific racism, followed by an in-depth debriefing with trained facilitators. A post-workshop survey was conducted to evaluate this anti-racism simulation workshop and assess the impact on participating occupational therapists (OTs) and physiotherapists (PTs). Following each simulation workshop, participants were invited to complete an anonymous post-activity survey (*n* = 20; 50% OTs, 45% PTs). Open text responses were analyzed thematically from the perspective of critical race theory.

**Results:**

The majority of the participants self-identified as women (95%); white (90%); mid-career (52%); and had never personally experienced racism (70%). All participants agreed that the workshop gave them ideas on how to start dismantling racism in their workplace. Thematic analysis resulted in four themes: so much to unlearn, remain humble, resist the silence, and discomfort is okay.

**Discussion:**

Despite feelings of discomfort, OTs and PTs appreciated anti-racism skills-based training and recognized the importance of taking action on racism in the workplace. Findings from this study support online (synchronous) anti-racism training as a viable and effective means of creating space for rehabilitation professionals to lean into brave conversations that are necessary for developing strategies to address racial microaggressions impacting Indigenous persons in the workplace. We believe that these small steps of preparing and practicing anti-racism strategies among rehabilitation therapists are essential to achieving a collective goal of dismantling racism in the health system.

## Introduction

Black, Indigenous, and people of colour continue to experience poorer healthcare and health outcomes (1–5). Indigenous-specific racism is a Canadian health crisis according to the National Collaborating Centre for Indigenous Health (6). Discrimination rooted in racism is a painful reality for many Indigenous peoples seeking health care in Canada (6). This can be explained by the long-term sequelae of racism, colonization and associated implicit biases of healthcare providers resulting in lower quality care (7, 8).

Racism can be understood on three different levels: *institutionalized*, *personally mediated*, and *internalized* (11). *Institutionalized racism* refers to differential access to goods, services, and opportunities based on race; *personally mediated racism* refers to both intentional and unintentional prejudice (assumptions) and discrimination (actions) based on race and; *internalized racism* as acceptance by racialized individuals of negative inferences about their own self-worth and abilities (11).

One way in which racism negatively impacts patient care is through microaggressions. Racial microaggressions are commonly occurring indignities, slights, or insults that send a message of derogatory or negative status to members of marginalized groups, intentional or not (9). These biases can be either overt or implicit, that is, the “unconscious collection of stereotypes and attitudes that we develop toward certain groups of people, which can affect our patient relationships and care decisions” (5). Where microaggressions are carried out by health care providers, therapeutic trust may be broken and quality of care may be impacted (10).

Canada's Truth and Reconciliation Commission (12) calls for “skills-based training in intercultural competency, conflict resolution, human rights, and anti-racism”, as well as trauma-informed care and addressing the gaps in health outcomes. Anti-racism response training is considered best practice in recognizing and addressing racism (13).

Rehabilitation is far from exempt from such experiences of racism (14–18). In order to develop strong anti-racist practice, providers need to be able to recognize racism and be prepared to respond in authentic and reflective ways (19, 20). To this end, an innovative educational workshop was developed for post-licensure learners using a simulation teaching and learning strategy. Simulation can provide the learner an opportunity to acquire and improve skills and behavior, which may lead to improved health services delivery (21). The purpose of this study was to evaluate the impact of an anti-racism simulation workshop with occupational therapists (OTs) and physiotherapists (PTs), as a strategy towards informing a practice of self-decolonization.

## Methods

This project was exempt from requiring ethics approval as this project fell under the category of program evaluation (Article 2.5 in the Canadian Tri-Council Policy Statement); no funding was received for this evaluation nor were survey respondents financially compensated for their participation.

### Simulation workshop description

A simulation workshop using live role-playing was created and delivered virtually to provide an opportunity to explore effective strategies in addressing racial microaggressions towards Indigenous persons in the workplace. Best practice standards in simulation (22) were followed, including a pre-brief, simulation and a comprehensive debrief by trained facilitators, as well as concepts of equity, diversity and inclusion (23). Actual workshop content was based on an original simulation design co-developed by two of the authors (DBD and MF) for entry-to-practice physiotherapy curriculum, and further refined collaboratively by all authors. Two of the authors self-identify as First Nations (DBD & PF), two identify as non-Indigenous academic researchers (MF & SO), two identify primarily as clinicians (AB & PF), and all but one are licensed PTs; all strive to practice critical allyship for action towards anti-racism.

Four 90-minute workshops were delivered virtually to more than 75 participants between December 2020 and November 2021. Workshop objectives included:

1. discuss the ongoing legacy of racism in Canada;
2. reflect on the role of addressing racial microaggressions in the healthcare setting, and;
3. enhance professional communication skills in addressing difficult, courageous conversations.

Following an introduction on racism and its impact on inequitable health outcomes among Indigenous peoples, practical strategies to address racism were reviewed with the participants. Strategies included breaking the silence with micro-resistant responses such as “What do you mean by that?”; responding to colour-blind statements (24) such as “why would you ever want to treat everyone the same and not embrace their individuality?”; and the “Opening The Front Door” technique (25), that is, *Observe*: Describe clearly and succinctly what you see happening; *Think:* State what you think about it; *Feel***:** Express your feelings about the situation, and; *Desire*: Assert what you would like to happen. The role of silence in implying tacit approval was discussed, along with avoiding additional emotional injury such as apologizing to the target after the racial microaggression or asking the target to fix the problem (25).

Part of the pre-brief of the simulation activity described included a warning that workshop participants should expect to feel uncomfortable, as they reflect on their own positionality and challenge their biases. Participants were then divided into small groups using virtual breakout rooms to apply the communication strategies in a fictional scenario. Trained facilitators played the fictional role of a senior colleague, “Mary”, who made racially-motivated disparaging remarks about an Indigenous client. Pre-scripted comments by the fictional colleague included, “you know they’re all alike; they just don’t know how to look after themselves” and “you’re not calling me a racist, are you?” Volunteers were asked to address the comments with their fictional colleague in their small groups using role playing. Role playing lasted for 5–10 min, followed by 10–15 min of small group debrief led by trained facilitators. Debrief questions included prompts such as: How did that make you feel and why do you think that is? Is this a realistic scenario? How could Mary's racist biases impact care and health outcomes?

### Workshop evaluation

All participants were requested to complete an anonymous electronic survey at the end of each workshop. Closed-ended questions were utilized to evaluate the workshop delivery format and open-ended questions to explore the impact on the participants. For example, “Did this workshop stimulate you to think differently about racism in the healthcare system? If yes, how?” (Supplementary Appendix S1).

Descriptive statistics using MS Excel were calculated for quantitative data while open text responses were thematically analysed from a lens of critical race theory. Critical race theory “can be used to understand the structural forces that drive racial inequities in society, and to work toward their dismantling” (26). Following the suggested guidelines for improving intercoder reliability (27), data was inductively analysed independently by two authors (MF & SO) with extensive experience in qualitative research. After sharing individual coding schemes, the two coders discussed the shared meanings of the codes and consensus was reached between the two coders on a framework of analysis. At this point, both coders returned to the data for repeat analysis from which no new codes were identified (27). The four final themes were shared with the other co-authors, who supported the results and no further refinements were made to any of the themes.

## Results

Close to one third of the participants completed the post-workshop survey (*n* = 22; response rate of 29%). Half (50%) identified themselves as OTs, 45% identified as PTs and 5% as “other”. The majority of the participants self-identified as women (95%); white (90%); mid-career (52%); and had never personally experienced racism (70%). All participants agreed that the workshop gave them ideas on how to start dismantling racism in their workplace: 100% of respondents either agreed (60%) or strongly agreed (40%) with the statements: “This workshop has clarified what role I can personally and professionally play in addressing microaggressions in the healthcare system” and “This workshop gave me ideas and tools on how I can start to dismantle racism in my workplace.”

Thematic analysis resulted in four themes: so much to unlearn, remain humble, resist the silence, and discomfort is okay.

### So much to unlearn

Participants spoke and wrote openly about the challenges of facing a history of colonization and racism in Canada that only recently they had been made aware of. As one respondent wrote, “I feel there is so much to unlearn!” [Participant (*P*) #7]. A different workshop participant stated, “Historical facts are key, but also reality right now from Indigenous perspectives. I have read a few books, attended several different education sessions through work and church, but there is much to learn!” [P#16]

Racism itself was not understood by all participants in its multi-layered complexity prior to the workshop as expressed by this individual. “I used to think of white privilege more so in terms of money and power vs. discrimination based on appearance and/or skin color”. [*P*#5]. While racism in the workplace may not always be overt and obvious, individuals expressed a readiness to acknowledge and face implicit racism head on. One individual wrote, “I think bringing it to the forefront is a good reminder of how much racism impacts us all…even if it's not always that obvious”. [P#1] Another wrote, “I think it (the workshop) reminded me that it (the health care system) is racist; reminders are important though because it's easy to become complacent, especially when I am not experiencing the racism”. [P#17]

During the workshop, participants spoke about the difficulty of having conversations about racism in the workplace, either with colleagues or with clients. As the same workshop survey respondent above stated, “white fragility is pretty rampant and has deep roots”*.* [*P*#7] Workshop participants recognized “how power differentials can affect interactions”. [#9]

Workshop participants were also asked to comment on any lingering emotional responses to the workshop on a personal level. One respondent wrote that the workshop content “continues to provoke thought as to how I fit within the systemic racism of healthcare and general society”*.* [*P*#12] Another wrote, “I want more! [P#14]”

### Remain humble

Respondents articulated the need to acknowledge the power they hold as healthcare professionals, while striving to learn more about their clients and ways to fight oppression. As this participant stated, “It (the workshop) served as a reminder of the immense impact we all have, as we work closely with others, clients and co-workers alike. For me most importantly, to remain humble and to always listen.” [*P*#1]

Participants recognized they were not experts in the lived experiences of others around them, and there was an opportunity for personal and professional growth. “I think the most important tools are education about true history and others’ perspectives”. [*P*#16]

Workshop participants were introduced to various communication tools and specific phrases they could use to disrupt racial microaggressions in the workplace. For many participants, actual phrasing of what they might say in a moment of potential confrontation with a co-worker was helpful but they admitted that initially, it might not be easy. As this person wrote, “I feel that using the tools and/or disruptive questions will take practice”.[*P*#3]

The realization that the work of dismantling racism is an on-going journey was articulated by this individual as follows. “I used to think that I did a good job at sticking up for others, and now I think I can do even more.” [*P*#9]

### Resist the silence

Workshop participants recognized the poignancy of responding to racism with their voices and not to stand by in silence. “Try to have the hard conversation” [*P*#2] as this participant wrote. They knew it was easier to say nothing, but that remaining silent would not move efforts forward towards dismantling racism, and would, in fact, perpetuate racism. “It is important for all of us to actively participate in improvement” [*P*#11] and “don't shy away from labelling it” [P#10].

Participants acknowledged the role they could and need to adopt if there is to be real change moving forward. They recognized the positive impact they could have by “ensuring that we do not contribute to it, perpetuate it, or to allowing it to continue” [*P*#9]. They accepted the fact that they need to “check the language you use from the perspective of others…is it hurtful to others and perpetuating white fragility?” [P#19]

When facing the authenticity of the scenario, participants found strength in their groups and felt compelled to speak up.

“My small group was really great and I could feel my anxiety rising when the facilitator playing Mary was so accurate in portraying this character. It was frustrating to hear these things, typical comments that we do hear day in and day out…I think they become so commonplace you almost tune them out…so I think hearing them said in this situation really made me wake up and go “wow, this really does happen this way’. I think it really reinforced the message with me to call this wrong behavior out.” [*P*#17]

It was also clear that participants were reflecting on how anti-racism training would benefit them not only in the workplace, but also beyond as evidenced by the following remarks.

“I think this (workshop) really helped me to clarify why it is so important to no longer be silent as it indicates passive agreement. I have even encountered it within my own family of extended relatives and don't say anything because I don't know what to say or how to confront it. Having the discussions we did in the breakout session really helped me to understand that when we stay silent, we are hurting the person on the receiving end…just as much as if we had instigated it ourselves…Before this workshop, I used to think that I wasn't participating in racism if I was silent or didn't give it any attention (that is, ignore bad behavior, don't draw more attention to it) and now I think when I remain silent I am hurting the person being targeted as much as if I had said it myself.” [P#19]

Before this workshop I used to think that I should let microaggressions go; and now I think I should try my best to address them, even with a microresponse.[*P*#17]

As this participant stated,

“Before this workshop, I used to think there's not much I could do systemically to change this type of rhetoric; and now I think I might be correct, unless I get REALLY loud and courageous.” [*P*#12]

### Discomfort is okay

A continuum of skill and confidence in having these conversations was observed by the facilitators, regardless of the years of professional experience of each participant. Not all individuals volunteered to participate in the role play activity, preferring to observe. Finding the appropriate words required courage and taking risk. As this participant stated, “I felt unsettled afterwards and did a lot of reflecting.” [*P*#15] Participants described their own emotional responses to the scenario and for some, their desire to be ready to respond. “Racist comments tend to trigger me and I get angry and irritable. I would like to be more mindful of my responses so as to be effective with my intentions.” [*P*#7]

Participants described accepting the feeling of discomfort, despite not always having the definitive response ready at hand.

“I learned so much from this diverse panel on how to begin these conversations and feel ok with being uncomfortable. Knowing that we all have to begin these conversations and trust that we don’t always know the right answer and that's ok.” [*P*#20]

Leaning into this discomfort was exemplified by one respondent as follows.

“It (the workshop) gave me a bit more courage to call out things that are not right. I am often hesitant for a number of reasons to do this, but this experience has helped me feel more confident in how I approach these situations…While having conversations with people in this regard is difficult and uncomfortable, these conversations take courage and are worthwhile; I want the system to change so I need to be courageous…” [*P*#17]

## Discussion

Recent literature reviews suggest that multiple strategies exist for education in antiracism (28–31), but there has been little rigorous evaluation of the actual teaching methods and impact on the learners (29, 31). This evaluation provides the input of post-licensure learners on anti-racism training using simulation to address racial microaggressions in the workplace and beyond.

Participants in this anti-racism simulation workshop voiced appreciation for skills-based training, as uncomfortable as they may have felt in the moment of addressing racism or during the activity of role-playing. Individuals reported being reluctant to call someone a “racist” or use the term “racism”. They were very conscious of potentially disrupting collegial relationships even though they acknowledged that quality care could be impacted when saying nothing. This finding is consistent with the anti-racism response training literature (13). A teaching and learning environment that embraces “brave classrooms and courageous conversations” (32) must be established that challenges our assumptions, beliefs and biases, where risks are taken for the benefit of learning. While no facilitators can ever guarantee a universally safe space for all teachers and learners, efforts must be made at creating safer spaces for all to engage in courageous conversations.

Psychological safety has been defined as “a shared belief that the team is safe for interpersonal risk taking” (33). It has also been described as a space where learners feel safe to be uncomfortable (34). All facilitators of this simulation workshop had explored their own positionality of privilege and experience of racism extensively prior to the workshops, but only some had experience as educators. It has been reported elsewhere that education for addressing cultural humility and racism requires trained and knowledgeable faculty (30, 35), but this does not negate the emotional impact the experience may have on the facilitators themselves. Following each simulation workshop, the facilitators held their own debrief session to unpack and navigate their own responses to the discussions on racism that took place. This important step has been referred to as *debrief the debriefing* (36)*.* The support and insight that the facilitators were able to offer one another was critical to the ongoing adaptation and success of the workshop series.

In the context of psychologically safe learning environments in simulation, three defining attributes have been identified (37): (1) The ability to make mistakes without consequences; (2) the qualities of the facilitator, and; (3) foundational activities such as orientation, preparation, objectives and expectations. A psychologically safe learning environment in simulation is described as “a feeling or climate whereby the learner can feel valued and comfortable yet still speak up and take risks without fear of retribution, embarrassment, judgment or consequences either to themselves or others, thereby promoting learning and innovation” (*p*. 49). Contrary to this definition, authentic and meaningful ways of addressing racism in simulation should not be expected to keep learners (and facilitators) feeling “safe” as participants lean into and embrace feelings of discomfort as they reflect on their own positionality. As has been argued elsewhere, true learning only occurs when we are at the edge of discomfort (32). Workshop participants acknowledged this discomfort and recognized this emotional response was necessary to be part of the solution in explicitly and consciously addressing and redressing racism. Individuals need to be comfortable with their discomfort; to talk about privilege and ways to use privilege to redress racism and discrimination; practice engaging in anti-racism discourses and activities; to hold systems accountable in dismantling racism, and; respectfully engage Indigenous communities in responding to racism (38). Notwithstanding the need to engage with Indigenous partners, Indigenous persons should not be the ones who are burdened with this work. Dismantling Indigenous-specific racism is the responsibility of those who sustain and benefit from the structures and systems that uphold this type of racism.

“Allyship” in the context of anti-oppression has been defined as “a lifelong process of building relationships based on trust, consistency, and accountability with marginalized individuals and/or groups of people” (39). Similarly, the goal of “critical allyship” refers to working in solidarity with people who are disadvantaged by structures and systems of society that uphold inequities (40). This critical allyship is necessary to guide actions of people in power and privilege to resist unjust structures that perpetuate health inequities (40). Alternatively, “structural competency” (41) is the learned ability to recognize health issues as implications of upstream administrative decisions, attitudes, biases, systems and structures. Recognition of structural forces can lead to an awareness of a host of environmental, systemic, attitudinal and contextual factors impacting health and wellness. Advancing this concept further has been a call for anti-racism as a professional competence, where healthcare professionals must act in solidarity with affected communities to advocate for structural improvements (42).

Along the continuum of competence training, anti-racism competence can also be explored through the lens of Indigenous ways of knowing, such as restorative justice practices (43). Restorative justice utilizes a collaborative decision-making approach that includes individuals who have been harmed and perpetrators who have caused intentional or unintentional harm. The process includes holding offenders accountable by having them accept and acknowledge responsibility for racist behavior, by repairing the harm they caused and to reduce the risk of re-offense by building positive relationships. This unique approach to implementing restorative practices can ensure fair treatment of all, protect the climate of the work or learning environment, and boost morale and trust while minimizing institutional liability. Healthcare providers trained in restorative justice practices would be well positioned to assist others to behave in ways based on humility, while responding sincerely and effectively to racist attitudes and behaviors by others.

## Limitations

The survey response rate was approximately one third of the 75 individuals who participated in this anti-racism simulation workshop. While a 29% response rate provides valuable information, it is not known why the response rate was not higher. It can be speculated based on the small group discussion and debriefing that participants reacted emotionally to the workshop and may have needed to sit in their silence while navigating a mixture of feelings at the conclusion of the workshop. The option to complete the workshop evaluation at a later date was not provided, something our team will consider in future workshop sessions.

This study was not intended at the outset to be a rigorous qualitative study. We did not explore the impact of the virtual teaching and learning environment compared to an in-person environment, nor the lived experiences of the facilitators.

It must also be acknowledged that the survey questions were written and responses analysed by two qualitative researchers who themselves bring their own colonial settler biases and perspectives. Efforts were made to counter any colonialist settler biases through repeated discussion by the collective group of authors on their personal experiences and emotive responses following each workshop.

## Conclusions

This program evaluation provides evidence that virtual (synchronous) anti-racism response training provides a viable and effective means of leaning into brave conversations for OTs and PTs. Although the case that was utilized was based in racial microaggressions towards an Indigenous person by a colleague in a healthcare setting, there are elements of the simulation activity that are transferrable to different contexts and settings. It is clear that anti-racism workshops are one small step forward to taking action against racism in the workplace that provide hope for mitigating the harms of racism in the health system in the future.

## Data Availability

The raw data supporting the conclusions of this article will be made available by the authors, without undue reservation.
