# Supplementary material for: Addressing racism in the workplace through simulation: So much to unlearn
Source: Front Rehabil Sci. 2023 Mar 30;4:1126085. doi: 10.3389/fresc.2023.1126085 (PMC10097889; doi:10.3389/fresc.2023.1126085)
Supplement: Supplementary file 1 [file Datasheet1.pdf]

Thank you

Please take a few minutes to answer the following post-workshop questions.

1. Having completed this virtual workshop, I have a better understanding of the impact of racism in the healthcare system.

- ◯ Strongly Agree
- ◯ Agree
- ◯ Neutral
- ◯ Disagree
- ◯ Strongly Disagree

2. This workshop has clarified what role I can personally and professionally play in addressing microaggressions in the healthcare system.

- ◯ Strongly Agree
- ◯ Agree
- ◯ Neutral
- ◯ Disagree
- ◯ Strongly Disagree

3. This workshop gave me ideas and tools on how I can start to dismantle racism in my workplace.

- ◯ Strongly Agree
- ◯ Agree
- ◯ Neutral
- ◯ Disagree
- ◯ Strongly Disagree

4. Did this workshop impact you emotionally and spiritually? Yes; No; If yes, how?

5. Did this workshop provide you with tools for action against racism? Yes; No; If yes, how? If not, what else would you need to take action against racism?

6. Did this workshop stimulate you to think differently about racism in the healthcare system? Yes; No; If yes, how?

7. What else would you like to learn about this topic?

8. What was the single biggest takeaway for you from this workshop?

9. Please complete this sentence: Before this workshop, I used to think ( ); and now I think ( ).

10. Please feel free to share any other final comments you have with the organizers of this workshop.

11. Please identify your profession.

- ◯ Occupational Therapist
- ◯ Physical Therapist
- ◯ Other (please specify)

12. How many years have you been in practice ?

- ◯ first 5 years of practice
- ◯ 6-10 years of practice
- ◯ 11-20 years of practice
- ◯ 20-30 years of practice
- ◯ >30 years of practice
- ◯ I am not a physiotherapist

13. Which of the following most accurately describe(s) you? (choose as many as you like)

- [ ] woman
- [ ] man
- [ ] two-spirit
- [ ] gender fluid
- [ ] non-binary
- [ ] transgender
- [ ] not sure/questioning
- [ ] prefer not to say
- [ ] another gender identity. Please specify.

14. Have you personally ever experienced racism?

- ○ No, never
- ○ Yes, once or twice
- ○ Frequently over my lifetime
- ○ Several times a week

15. Please identify which race you most strongly identify with.

- ○ White
- ○ Black or African Canadian
- ○ Hispanic or Latino
- ○ Asian or Asian Canadian
- ○ Indigenous
- ○ Another race
- ○ I prefer not to say
